# The Use of Cellular Technologies in Treatment of Liver Pathologies

**Published:** 2012

**Authors:** O.S. Petrakova, E.S. Chernioglo, V.V. Terskikh, E.N. Kalistratova, A.V. Vasiliev

**Affiliations:** Koltzov Institute of Developmental Biology, Russian Academy of Sciences, Vavilova Str., 26, Moscow, Russia, 119334; Faculty of Biology, Lomonosov Moscow State University, Leninskie Gory 1/12, Moscow, 119991

**Keywords:** cell transplantation, cellular therapy, differentiation, liver

## Abstract

Cell techniques find increasing application in modern clinical practice. The II and III phases of clinical trials are already under way for various cellular products used for the restoration of the functions of the cornea, larynx, skin, etc. However, the obtainment of functional cell types specific to different organs and tissues still remains a subject of laboratory research. Liver is one of the most important organs; the problems and prospects of cellular therapy for liver pathologies are currently being actively studied. Cellular therapy of liver pathologies is a complex multistage process requiring a thorough understanding of the molecular mechanisms occurring in liver cells during differentiation and regeneration. An analysis of the current cellular therapy for liver pathologies is presented, the use of various cell types is described, the main molecular mechanisms of hepatocyte differentiation are analyzed, and the challenges and prospects of cell therapy for liver disorders are discussed in this review.

## INTRODUCTION

The treatment of liver diseases is a significant problem of modern medicine. The statistical data tell us that more than 200,000 people are diagnosed with various chronic and acute liver diseases in the Russian Federation annually. Despite the progress achieved in modern medicine, conventional therapeutic approaches remain insufficient for treating chronic and acute liver pathologies; the mortality rate thus remains at the level of 80–90%.

Transplantation of liver or its parts remains the major method for treating severe pathologies. The shortage of donor material has spurred an active search for approaches of cell therapy for liver diseases. A large body of data accumulated over recent years attests to the fact that cell therapy can be considered as one of the priority areas in modern biomedicine and biotechnology.

Cell therapy has a number of significant advantages:

1. As opposed to sophisticated surgery, cell transplantation is technically a much simpler and less invasive procedure; it has no risk of rejection or other complications.

2. Donor material for cell therapy is easier to obtain; it can be prepared beforehand and cryopreserved for long-term storage.

3. Cell transplantation not only compensates for the organ dysfunction and facilitates restoration of the function of a patient’s own cells, but it also impedes the emergence of fibrosis in damaged tissues by filling the missing cell niche.

4. The cells, upon autologous transplantation, are not eliminated by the immune system and can give a prolonged (or permanent) effect. In the case of allogeneic transplantation for inherited disorders, the donor material can compensate for the recipient’s genetic defect as normal proteins are synthesized by donor cells.

The efficiency of substitution of tissue defects, ability to stimulate a recipient’s own organ repair, the absence of a risk of emergence of fibroses mainly depend on the cells being used. It has been demonstrated in a number of studies that cells of different types can express hepatocyte-specific markers under certain growth conditions. However, the true functionality of particular cells still needs proof. The question that emerges is what criteria does a transplanted cell need to meet in order to provide efficient compensation for the dysfunction of the damaged liver? Firstly, that would be the ability to carry out synthetic and detoxication functions. The cells need to be capable of expressing hepatocyte-specific proteins, such as cytochromes P450 and albumin, as well as storing glycogen, synthesizing urea, binding bilirubin, etc. The search for the optimal cell sources and obtainment of functionally active types of cells in amounts sufficient for transplantation obviously remain among the main challenges of cell biology. The cells need to be easy to obtain and capable of rapid *in vitro* proliferation, endure long-term cryostorage, be immunocompatible and capable of differentiating into functionally active hepatocyte-like cells.

**Fig. 1:**
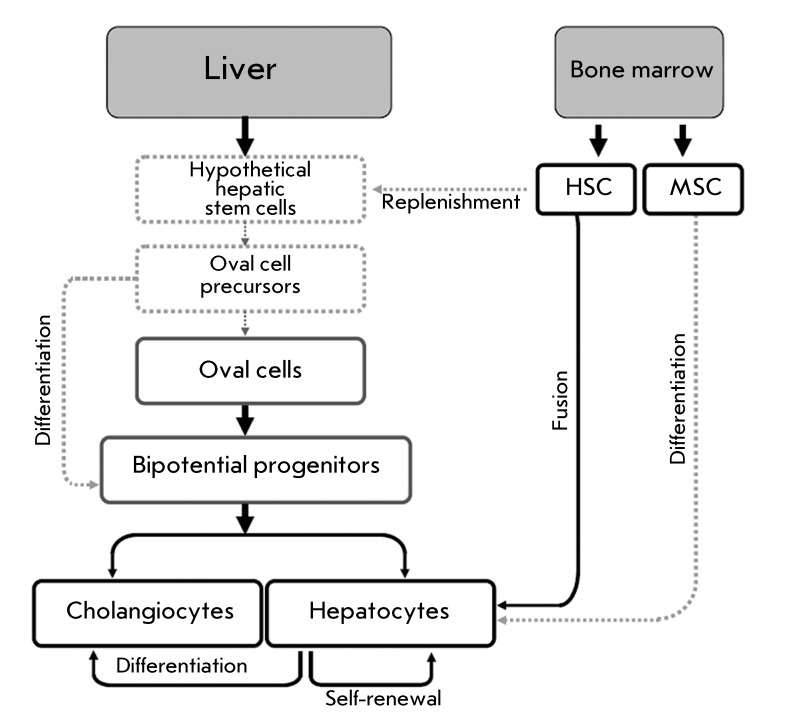
Mechanisms of cellular regeneration of postnatal liver. Taken and modified from [2, 3]. The scheme is hypothetical.

Repair success also depends on participation of the growth factors, cytokines and chemokines, which are part of the complex signalling system coordinating cell behavior. For this reason, the cells capable of identifying the proper growth factor combination can be proposed for the stimulation and correction of the repair of certain tissue defects. On the other hand, the cells being used may make a significant contribution (in many cases, the contribution is crucial) to the repair process due to transdifferentiation into target-differentiated and functional-tissue cells.

## MEChanisms of liver cell regeneration

The liver possesses a high degree of self-restoration and a considerable capability of repair even after resection of its largest part. These properties are provided by a complex regeneration system ( *Fig. 1* ). Its major features include the proliferative capability of differentiated hepatocytes, as well as their ability to produce mature hepatocytes and transdifferentiate into cholangiocytes [1]; regeneration from the reserve stem cells; repair with haematopoietic cells via fusion of myeloid cells with damaged hepatocytes and/or differentiation of bone marrow mesenchymal stem cells into hepatocyte-like cells [2, 3].

**Fig. 2:**
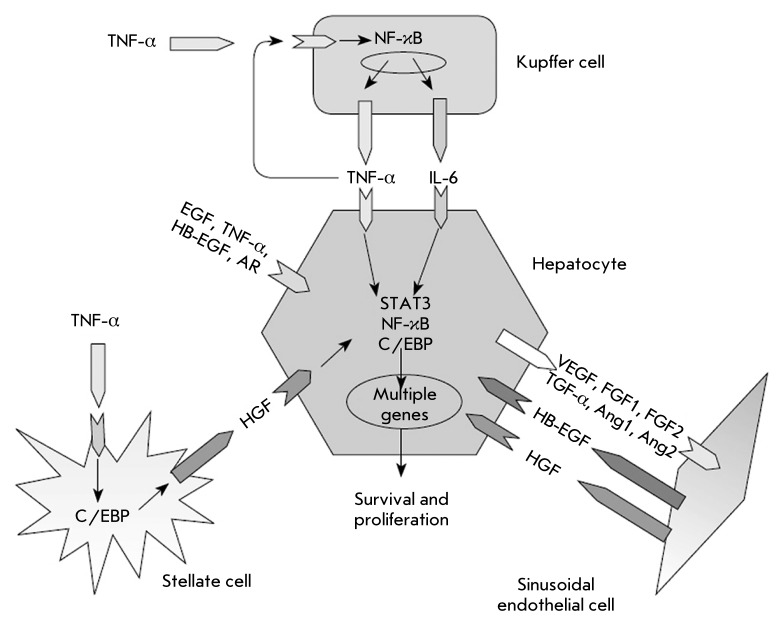
Molecular mechanisms of hepatocyte population maintenance and initiation of hepatocyte proliferation. Taken from [7].

Hepatocytes are differentiated polyploid cells; however, their capability to proliferate and population maintenance makes them similar to stem cells. In adult liver, hepatocytes mostly remain in a dormant state (G0 phase of the cell cycle); however, if regeneration becomes necessary, hepatocytes start dedifferentiating, proliferating, and reproducing differentiated hepatocytes. For example, after biliary cells in rat liver were damaged, hepatocytes exhibited a certain degree of phenotypic plasticity and were capable of transdifferentiation into cholangiocytes [1]. The hepatocyte population increases without the participation of stem cells during the postnatal growth [4]. During the fetal and early postnatal periods, hepatocytes undergo mitosis, followed by the process of mitotic polyproidization, resulting in an increase in the number of hepatocytes and their ploidity. Cytotomy does not occur in the first cycle after DNA replication, giving rise to a binuclear hepatocyte. The next mitotic cycle after DNA duplication includes synchronous nuclear division; chromosomes aggregate to yield a single mitotic plate, giving rise to two mononuclear tetraploid cells. The alternation of these two cycles with a gradually increasing hepatocyte ploidity occurs subsequently [5]. In order to make possible postnatal growth of the liver, the initially diploid hepatocytes undergo five or six polyploidizing mitoses. However, in the cases requiring rapid regeneration (e.g., after exposure in toxic or infectious conditions, etc.) mitoses without cytokinesis are temporarily eliminated and cell fission proceeds via the conventional pathway. This protects liver cells against excessive polyploidization. The major factors regulating hepatocyte proliferation in liver regeneration include interleukin-6 (IL-6) and the tumor necrosis factor α (TNF-α) secreted by Kupffer cells, as well as the hepatocyte growth factor (HGF) secreted by stellate cells. These factors initiate hepatocyte transition from the G0 to the G1 phase. The transforming growth factor β (TGF-β) suppresses the entrance of hepatocytes into mitosis upon completion of regeneration. HGF, the vascular endothelial growth factor (VEGF), and the fibroblast growth factors 1 and 2 (FGF1, FGF2) secreted by endothelial cells play an important role in the replication and viability maintenance of hepatocytes as well [6, 7]. The major molecular mechanisms making possible hepatocyte proliferation are schematically shown in *Fig. 2* .

**Fig. 3:**
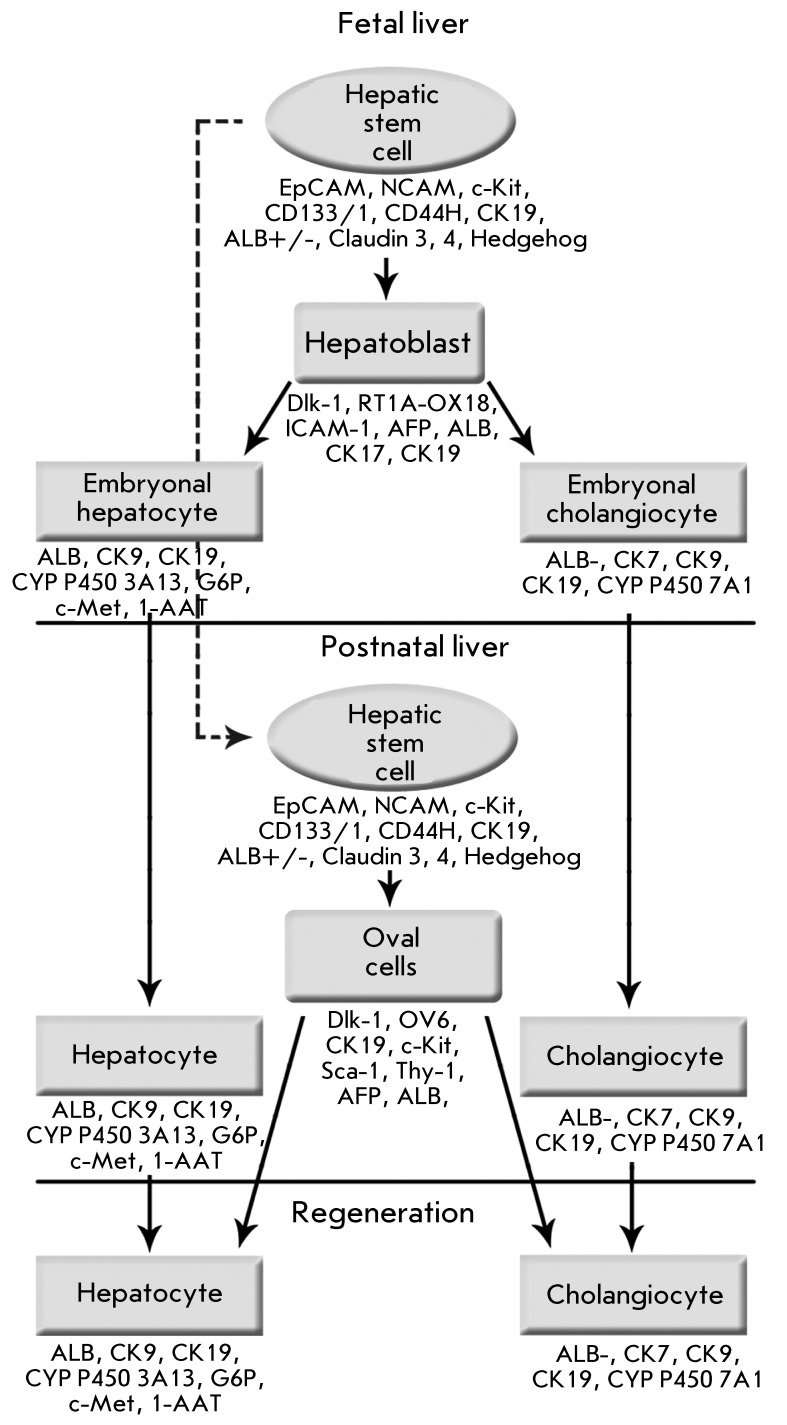
The hierarchy of liver stem cells. Taken and modified from [2, 115]. The scheme is hypothetical.

Hepatic stem cells also play a significant role in the regeneration process if the hepatocyte population proves incapable of repairing the damaged liver (after the resection of the critical part of the organ, upon extensive toxic, infectious, etc. lesions). The postnatal liver contains a number of stem cells whose hierarchical relationship is still under discussion [8]. Oval cells are the major precursors of hepatocytes and cholangiocytes. The term “oval cells” is usually used to refer to a population of small cells (about 10 µm) that possess bipotent differentiation potential and are characterized by a high nuclear-cytoplasmic ratio. Oval cells presumably originate from the canals of Hering, which are believed by some authors to exclusively consist of stem cells [9]. Oval cells express albumin, α-fetoprotein, cytokeratin 19, the specific surface marker OV6 (А6 in mice), and the embryonic marker Delta-like/Pref-1 that is also typical of hepatoblasts [10]. In addition, oval cells produce stem cell markers, such as c-Kit, Sca-1, nestin, and CD90 (Thy-1). In all likelihood, the population of these cells is heterogeneous and may contain cells of different origins. Some cells carry the CD45, c-Kit, CD90 markers and albumin. These cell populations presumably consist of haematopoietic stem cells that penetrate into the liver from the blood flow [11]. In general, the population of true oval cells expressing the markers OV6 and cytokeratin 19 is the population of committed, temporarily proliferating hepatic stem cells. An assumption was made that the adult liver has a compartment with less differentiated cells, the original stem cells of the postnatal liver. A population of stem cells expressing the epithelial cell adhesion molecule EpCAM was obtained in [12]. These cells were referred to as hepatic stem cells EpCAM ^+ ^ (hHpSCs); in the fetal liver, they act as hepatoblast precursors; in the postnatal liver, they reside in the canals of Hering. Hepatic stem cells also express NCAM, c-Kit, CD133/1, CD44H, cytokeratin 19 and are weakly positive with respect to albumin. Hepatic stem cells do not express α-fetoprotein, CD45, or mature hepatocyte markers (cytochromes Р450, intracellular adhesion molecules ICAM-1, transferrin). With *in vitro* differentiation induced, the cells proved capable of synthesizing α-fetoprotein and ICAM-1. Transplantation of hepatic cells to NOD/SCID mice induced the synthesis of proteins typical of mature hepatocytes (albumin, transferrin). It was assumed that these cells are stem cells in the fetal and postnatal liver and may presumably be precursors of oval cells [12]. The general hierarchy of hepatic stem cells is shown in *Fig. 3* .

Bone marrow stem cells can also contribute to liver regeneration. The liver is known to serve as a haematopoietic organ during the fetal and early postnatal periods. In the adult liver, part of the population of oval cells is made up of haematopoietic cells that are CD34-, CD45-, and CD133-positive; the liver can become an organ of extramedullary haematopoiesis upon certain pathological processes. It has been demonstrated that if bone marrow from male mice is transplanted to lethally irradiated female mice, 1–2% of hepatocytes carry the Y-chromosome marker 6 months following the transplantation. These hepatocytes express albumin and can be either diploid or polyploid [13]. When studying the biopsy material obtained from the liver of six women who received haematopoietic cells obtained from the peripheral blood of male donors, the Y-chromosome was revealed in hepatocytes at an s frequency varying from 0 to 7% [14]. Haematopoietic stem cells were assumed to be capable of differentiating into hepatocytes; however, a number of studies have demonstrated that haematopoietic cells can fuse with a recipient’s hepatocytes, thus preventing their death and stimulating regeneration [15, 16]. Myelocytary cells, granulocytes, and macrophages/monocytes also undergo fusion with hepatocytes [16]. The relative contribution of transdifferentiation and cell fusion to liver repair by haematopoietic stem cells is currently being discussed. There is a possibility that both these processes occur in the organism.

## use of cells isolated from donor livers

Hepatocyte transplantation can serve as an alternative approach to the liver transplantation that is conventionally used in modern clinical practice. It is a commonly known fact that liver transplantation may include the substitution of either the entire liver with a donor organ or part of it. However, the shortage of donor organs, the poor implant survival rate, and significant complications due to rejection or insufficient functioning of the transplanted liver limit the applicability of this method to a significant extent. Furthermore, a sufficiently efficient procedure enabling long-term storage of the liver as a whole organ has not been elaborated thus far. Due to these reasons, transplantation of hepatocytes isolated from a donor liver becomes a promising direction of cell therapy for liver disorders. The advantages of this approach include the possibility of using both freshly isolated cells and cells subjected to long-term cryostorage; donor cells can compensate for the pathologies caused by genetic disorders and act as gene therapy vectors. Hepatocyte transplantation is a significantly less invasive procedure; it virtually has no risk of rejection. The transplanted hepatocytes fill the cell niches that remain empty as a result of mass death of the patient’s own cells (e.g., after acute exposure to toxic or infectious conditions), which considerably reduces the risk of fibrosis formation. Moreover, hepatocyte transplantation does not require resection; thus, regeneration of the patient’s own organ is possible (e.g., upon acute hepatic failure).

The hepatocyte transplantation procedure includes a number of conventional techniques elaborated in accordance with the GMP (Good Manufactured Practice) requirements [17]. A donor liver that cannot be used for transplantation due to fatty dystrophy (over 40–50% of the organ), chronic ischemia, mechanical damage, liver capsule rupture, blood group mismatch, damaged blood vessels or biliary ducts can serve as a hepatocyte source [18–20]. Fetal liver can be used for transplantation in rare cases [21]. The cell sources may include the liver from non-heart-beating donors, liver affected with atherosclerosis or fibrosis. The standard hepatocyte isolation procedure includes liver perfusion, enzymatic treatment to disintegrate the intercellular substance, and washing of the resulting cell suspension. The isolated hepatocytes are typically characterized by an approximately 70–90% viability and (1—17) х 10 ^6^ cells/g of tissue (hepatocytes with at least 60% viability are recommended for use for clinical purposes). The cells obtained are cooled to +4°С and immediately re-suspended in an infusion solution to be directly transplanted or in a freezing solution for subsequent cryostorage [22, 23]. The metabolic characteristics of the hepatocytes are checked based on the activity of the cytochromes Р450 (CYP1A2, CYP2A6, CYP3A4, CYP2C9, and CYP2E1) and their ability to synthesize urea [24].

Hepatocytes are typically transplanted via the portal vein, the splenic vein, or via an intraperitoneal catheter. Direct transplantation into the peritoneal cavity, pancreatic gland, or hepatic parenchyma demonstrates a poorer survival rate of hepatocytes. Introduction via the portal vein is regarded as the best method of transplantation; however, when performing this procedure, one needs to control the pressure in the portal vein to prevent its obstruction [25, 26]. Introduction of hepatocytes into the spleen is typically used in patients with chronic liver disorders, when fibrosis impedes cell engraftment. The amount of cells required for transplantation depends on the type of pathology and is equal to about 5–10% of the theoretical liver weight ((2–4) х 10 ^8^ cells/kg of body weight); however, no more than 1% of the amount of a patient’s hepatocytes is introduced per procedure. The adult human liver contains approximately 2.8 x 10 ^11^ hepatocytes; therefore, the recommended amount of donor cells to be introduced per transplantation procedure is (2–4) x 10 ^9 ^ [27]. According to some reports, the amount of cells to be transplanted can be lower in case of chronic disorders, whereas it needs to be increased for the therapy of inherited pathologies. A stable therapeutical effect is achieved on week 4–8 following transplantation and lasts for 6–9 months.

At the moment of writing, donor hepatocytes have been transplanted to more than 80 patients in 13 medical centres [18, 19, 28–30]. Among them, about 30 (including children) had inherited metabolic disorders of the liver, such as ornithine transcarbamylase deficiency or glycogenosis. Hepatocyte transplantation significantly improved the condition of patients with inherited disorders. It has also been demonstrated that hepatocyte transplantation can stabilize the condition of children awaiting donor liver transplantation [29, 31]. In a series of case reports, e.g., in patients with the Crigler-Najjar disease, the amount of cells required to achieve a stable clinical effect is equal to 12% of the patient’s liver weight; therefore, repeated transplantations are needed because of the limited amount of cells that can be introduced per transplantation. Hepatocyte transplantation in patients with disorders of bilirubin metabolism can be a successful alternative to whole liver transplantation during a period of over 11 months [32–34]. Restoration of normal glucose levels has been observed in patients with glycogenosis (both children and adults) [19, 35].

The major drawback of this method is a shortage of donor material. The priorities in this field include the improvement of the quality of the isolated hepatocytes, optimization of cryostorage procedures, and enhancement of the efficiency of liver “accommodation.” No optimal immunosuppressive procedures have been designed thus far as well: the transplanted donor hepatocytes are known to be eliminated from the liver in 6–9 months. Approaches may include selecting optimal populations of hepatic stem cells capable of proliferation and significant *in vitro * division followed by differentiation, and designing proper cell lines [36]. On the other hand, the search for an optimal alternative source of cells (including authologous sources) for the therapy of liver disorders remains a priority.

## alternative sources of cellULAR material

The demand for alternative sources of cellular material for the therapy of liver disorders is mainly fuelled by the shortage of donor organs and low availability and insufficient amount of hepatocytes that can be used for transplantation. Moreover, cells obtained from alternative sources can be used for autologous transplantation. It has been demonstrated that different cell types are to a certain extent capable of differentiating into hepatocyte-lineage cells; however, no functionally active hepatic cells have been obtained thus far [37]. Embryonic stem (ES) cells and induced pluripotent stem (iPS) cells [38–41], as well as hepatic stem and progenitor cells, [12, 42] are the best studied both experimentally and clinically at this moment. Mesenchymal cells from bone marrow [43, 44] and adipose tissue [45–47], amniotic fluid cells [48–50], etc. have been studied as cells capable of differentiation into hepatocytes. However, only partial transdifferentiation has been observed in these studies; the functionally active state that is typical of hepatocytes has not been attained.

**Pluripotent ES and iPS cells**

**Table 1:** The major stages of differentiation of ES cells into hepatocytes [55, 56]

Differentiation stage	Duration, days	Major differentiation markers	Hepatocyte markers characteristic for this stage
Induction of endoderm formation	3–4	Activin A	Sox17.Hnf-3β
Cell commitment to the hepatocyte lineage	4–7	BMP2, FGF4	Hnf-3β,alpha fetoprotein
Proliferation of hepatoblast-like cells	5–10	HGF, KGF	Albumin, alpha fetoprotein, G6P, TAT
Maturation of hepatoblast-like cells	8–15	Oncostatin M, dexamethasone, N2, B27	Albumin, G6P, TAT, PEPCK, TDO, CYPP450, etc.

The interest in embryonic stem cells is mainly rooted in their broad differentiation potential: embryonic stem cells isolated from the inner cell mass of blastocysts retain their pluripotent properties upon long-term *in vitro* cultivation and can produce cells of all three germ layers. At the time of writing, a large amount of studies have been devoted to the differentiation of ES cells into various cell types of the adult organism. Meanwhile, the practical use of ES cells can be limited by a number of unsolved problems, such as the risk of teratoma formation, ethical issues related to the destruction of embryos, long-run and labor-intensive differentiation protocols, etc. The low immunogenicity of human ES cells has been reported, which may also be of interest. However, it remains unclear whether these cells retain their low immunogenicity after differentiation into a certain lineage is induced [51].

The hepatocyte differentiation protocols of ES cells include several major stages imitating the processes occurring during liver development [52–54]. The major stages of the process are given in *Table* .

Various demethylating agents are used to enhance the differentiation efficiency. The idea of using demethylating agents is based on their ability to activate gene expression by DNA demethylation: demethylation of the promoter regions activates gene expression, which significantly broadens the differentiation potential of cells. However, since DNA demethylation is a random process, the combination of demethylating agents and growth factors or cytokines is used to commit cells into a certain lineage [32]. The differentiation efficiency of murine ES cells was successfully increased using valproic acid inhibiting histone deacetylase [57]. Hepatocyte-like cells capable of synthesizing albumin, cytochromes P450 and accumulating glycogen have thus been obtained. Differentiation without valproic acid yielded structures resembling biliary duct cells. However, in this case, the injection of ES cells differentiated into the hepatocyte lineage to Balb/c nude mice resulted in teratoma formation [57]. It should be mentioned that no teratomas have been observed after human ES cells differentiated into hepatocytes are injected to immunodeficient mice, whereas the injection of undifferentiated ES cells has resulted in teratoma formation [55, 58].

Another source of hepatocyte-like cells is iPS cells. iPS cells are induced pluripotent stem cells that are artificially obtained from the somatic cells of the human organism, into which certain genes and factors that are important to attain the pluripotent state are introduced [59]. Identically to ES cells, iPS cells can differentiate into cells of all three germ layers; however, opposite to ES cells, it is possible to obtain autologous iPS cells for substitutive cellular therapy and iPS cells from patients with various inherited disorders to simulate the pathological process *in vitro* and test therapeutic agents [60, 61].

In general, the hepatocyte differentiation protocols of iPS and ES cells are similar. *In vitro* differentiation of human iPS cells into hepatocyte lineage cells using cytokines and adenoviral vectors expressing the *Hex* gene, which plays a significant role in hepatocyte development, yielded hepatocyte-like cells expressing the endoderm markers Hnf-3β and Sox17, as well as albumin and cytochromes P450 [60]. It was also shown [54] that 60% of the cells start producing albumin and α-fetoprotein on day 7 of the differentiation of human iPS cells using the standard protocol; by day 20, the cells were capable of synthesizing urea (approximately 15% of the level of urea synthesis by hepatocytes) and storing glycogen [54], but the percentage of hepatocyte-like cells was low (about 10%). However, the absence of an oncogenic potential for using these cells has not been demonstrated.

**Somatic cells**

*Hepatic stem and progenitor cells. *Multipotent postnatal hepatic and progenitor cells can be an alternative source for cellular therapy. They actively proliferate * in vitro* (and/or *in vivo* ), enabling one to obtain significant amounts of such cells from a small bioptate. These cells retain viability for a considerably longer time period and better endure cryostorage compared to mature hepatocytes; furthermore, they are characterized by a lower immunogenicity. Hepatic stem cells are both capable of *in vivo* differentiation into hepatocytes and population maintenance; this fact may prolong the therapeutic effect of their introduction. Stem cells are already committed to hepatocytes and require no additional time-consuming differentiation procedures. The major problem impeding the widespread use of these cells is the shortage of donor material.

Specific attention is given today to methods consisting in the isolation of hepatic stem cells and searching for optimal cell populations possessing the highest regenerative potential. Hepatic cells carrying the surface marker and epithelial cell adhesion molecule EpCAM were isolated by continuous-flow fluorometry. The percentage of such cells in donors of all ages is 0.5–2.5% of the hepatic parenchyma cells. These cells can undergo over 150 *in vitro* passages and are positive with respect to cytokeratins 8, 18 and 19, CD133/1, CD44H, and weakly positive with respect to albumin. Hepatic cells do not express α-fetoprotein, adult hepatocyte markers (cytochromes P450), intracellular adhesion molecules ICAM-1, markers of haematopoietic (CD45) and mesenchymal cells (desmin, VEGFRe). After differentiation is induced, these cells acquire the capability of synthesizing α-fetoprotein and ICAM-1. Transplantation of hepatic EpCAM ^+ ^ cells to NOD/SCID mice has resulted in the formation of hepatic structures from human cells and in the synthesis of proteins that are typical of mature hepatocytes. Thus, it has been assumed that these cells act as stem cells of the postnatal liver and can be used for substitutive cellular therapy [12]. In another study, a Thy-1 (CD90)-positive cell population was isolated from the adult donor liver via immunomagnetic sorting. In all likelihood, this population was heterogeneous and contained cells that were positive with respect to markers of progenitor cells, namely, haematopoietic cells – CD34, stem cells – CD117, CK19, duct cells – CK14, and oval cells – OV6. The population of Thy-1-positive cells possessed a higher differentiation potential compared to that of the Thy-1-negative population and was capable of differentiating both into hepatocytes and duct cells. The functional activity of these cells is supported by the expression of HepPar 1 and human albumin after they are injected to immunodeficient mice [42]. The isolation of the so-called SP (side population) cells via continuous flow fluorometry can be considered as another approach. A number of types of stem cells were shown to contain the ATP-dependent ABC transporters responsible for the elimination of various cytostatics and drugs, whose activity results in the development of the multiple-drug-resistance phenomenon, from the cell. Dye Hoechst 33342 is one of the compounds eliminated from stem cells; the use of this dye allows one to sort unstained small cells (referred to as SP cells) on a continuous-flow cytofluorimeter. CD45- and Hoechst 33342-negative SP cells capable of colony formation upon *in vitro* growth have been derived from the human liver. Large cells containing a large number of granules, intracellular lipofuscin and, rather frequently, the ambiguous nucleus emerged in the colonies after 2–3 weeks of cultivation. The cultured cells were positive with respect to human hepatocyte markers: namely, HepPar, cytokeratins 8 and 18, cytochromes Р450 and albumin. Thus, SP cells isolated from an adult donor liver are capable of *in vitro* differentiation into hepatocyte lineage cells [62]/

*HSC and MSC obtained from bone marrow, cord blood and adipose tissue*. The interest towards bone marrow stem cells as a potential source of hepatocytes appeared in early studies carried out by Petersen *et al.* [63]. Donor cells were found in the liver of irradiated mice after transplantation of the bone marrow; these cells subsequently differentiated into hepatocyte-like cells. These experiments have cast doubt on the previous assumption that hepatocytes can be obtained exclusively from endodermal sources. It turned out that hepatocytes with a male karyotype could be detected in women transplanted with bone marrow derived from male donors [13]. It remains unclear whether hepatocytes are formed from bone marrow cells via transdifferentiation, fusion, or lateral gene transfer; this question remains a subject for discussion [64].

Haematopoietic stem cells (HSC) can be easily sorted based on CD31 and CD34 markers and isolated from the bone marrow, cord blood, or, in certain cases, from peripheral blood. It has been demonstrated that upon hepatic lesions, transplanted human HSC become capable of producing albumin-synthesizing cells in murine liver and repairing hepatic defects both via fusion [15] and without fusion with the host cells [65]. Yet, the cell fusion phenomenon has not been observed in bone marrow-derived mesenchymal stem cells (MSC) [66]. MSC derived from bone marrow, cord blood, and adipose tissue exhibit immunosuppressive and anti-inflammatory properties, can be easily grown *in vitro* , and synthesize a number of cytokines and growth factors capable of stimulating the repair of a patient’s own cells. Because of these properties, MSC are often regarded as a convenient cellular source for substitutive cellular therapy [67–69]. The condition of mice with acute hepatic failure induced by carbon tetrachloride was shown to improve after the transplantation of bone marrow MSC. A significantly higher survival rate of hepatocytes was observed in the experimental group compared to the control group, despite the fact that MSC engraftment had not occured by the time of the observation. The positive effect of MSC introduction is attributed to their stimulating and anti-inflammatory action [70]. Intact MSC from human cord blood were also introduced into fetal sheep liver; expression of human albumin was detected 56–70 days following the transplantation; the percentage of human cells in lamb liver varied from 2.6 to 12.5% [71].

MSC have been differentiated into hepatocyte-like cells in a number of studies. Expression of α-fetoprotein and albumin was achieved through treatment of MSC from human adipose tissue with HGF, oncostatin M, and dexamethasone [45]. In another study, a hepatocyte culture medium and a demethylating agent (20 µM 5-azacytidine) were used to differentiate rat adipose tissue-derived MSC into cells expressing albumin, α-fetoprotein, cytochromes Р450 1А1, and cytokeratins 18 and 19 [46]. These cells were also capable of synthesizing urea. *In vitro* hepatocyte differentiation could not be induced upon differentiation of MSC derived from human bone marrow using FGF4, HGF, and dexamethasone. However, the addition of the demethylating agent trichostatin A (1 µM) inhibiting histone deacetylase yielded epithelium-like cells expressing cytokeratin 18. The cells also synthesized albumin, and they were characterized by enhanced cytochrome P450 activity and urea secretion [43].

Thus far transplantation of bone marrow cells for the therapy of liver disorders has been performed on several occasions [72]. The granulocyte colony-stimulating factor (G-CSF) was used in some of the transplantations to immobilize patients’ own bone marrow stem cells and to stimulate liver regeneration without isolating bone marrow [73, 74]. Transplantation of autologous bone marrow-derived stem cells to 27 patients with chronic hepatic disorders or cirrhosis resulted in an increase in albumin secretion and a decrease in the bilirubin level [75–77].

Despite some degree of success in using bone marrow-derived stem cells in patients with liver diseases, the mechanism underlying their action remains unclear. The problems related to safety have not been solved, including those associated with possible MSC-induced fibrosis, which may worsen the course of the disease [78]. The impact of these cells on damaged liver and their mechanisms of action need elucidation prior to making any attempts at using them in clinical practice.

*Amniotic fluid cells. *Amniotic fluid contains a heterogeneous population of cells of fetal origin with stem cells positive with respect to mesenchymal markers (CD29, CD44, CD73, CD90, CD105), neutral markers (nestin, β-3-tubulin, NEFH), and certain pluripotency markers (Oct4, Nanog). These cells are of interest mostly due to their broad differentiation potential: they can undergo *in vitro * osteogenic, adipogenic, neutral, endothelial, hepatocyte, etc. differentiation [50, 79–82]. It has recently been demonstrated that amniotic fluid stem cells can express epithelial markers (keratin 19, keratin 18, and р63) simultaneously with the mesenchymal markers [83]. This fact has disproved the previous concept that amniotic fluid stem cells are MSC. Although the status of these cells is being actively discussed, an assumption can be made that the ability to form fibrous lesions upon introduction of amniotic fluid cells will be lower than that for cells of truly mesenchymal origin. The drawbacks of this cellular source include the low availability of these cells, the limited amount of donor material, and the requirement to collect cells at a certain stage of the pregnancy, which is not always possible.

The possibility of hepatocyte differentiation of amniotic fluid cells has been demonstrated. The cells were grown in matrigel- or collagen-coated plates in the presence of HGF, FGF4, insulin, oncostatin M, and dexamethasone. Cell morphology was altered by day 7 of differentiation: the cells acquired a polygonal shape without spikes. Synthesis of albumin, α-fetoprotein, Hnf-4α, and HGF receptor c-Met was observed on day 45. The level of synthesized urea increased from 50 ng/h per cell in the control culture to 1.21 х 10 ^3 ^ ng/h per cell in the differentiated culture [49]. The differentiation abilities of human bone marrow-derived MSC and amniotic fluid stem cells were compared. Cells were grown in collagen I coated plates in the presence of differentiating agents: days 0–2 – FGF4, days 3–5– HGF, days 6–18 – HGF + insulin-transferrin-selenite + dexamethasone and trichostatin A (histone deacetylase inhibitor). Morphological changes were observed in both cultures starting on day 7: the cells became rounder and polygonal in shape. The shape of amniotic fluid cells subsequently changed to that of epithelial cells in a more rapid and stable fashion. It was demonstrated by quantitative PCR that the original expression of hepatocyte markers, such as α-fetoprotein, albumin, cytokeratin 18, Hnf-1α, C/EBPα, and CYP1A1, was either negligible or absent in both cell cultures. The expression of these markers remained virtually unaltered at the initial stage of differentiation. However, at the stage of hepatocyte maturation expression of hepatocyte markers increased significantly; on day 14 of the differentiation, expression of all the markers in the amniotic fluid cell culture was considerably higher than that in the bone marrow-derived MSC culture. Expression of all markers, with the exception of α-fetoprotein, increased at the stage of hepatocyte maturation. Expression of α-fetoprotein reached a maximum by day 14 of the differentiation, followed by a decrease, whereas maximum albumin expression was observed by day 28 of the differentiation. Albumin expression in amniotic fluid cells was approximately 1.3 times higher than that in bone marrow-derived MSC. An immunophenotypic analysis revealed that the percentage of cells that are positive with respect to hepatocyte markers is reliably higher than that in the MSC culture. These cells were also capable of synthesizing urea and accumulating glycogen [50].

All these data attest to the high potential of using amniotic fluid stem cells in cellular therapy; however, a better understanding of their differentiation status and fibrosis formation ability is required.

*Cells of endodermal origin*. The possibility of cell transdifferentiation within the same developmental germ layer lineage is currently being actively studied. The advantages of this approach are obvious: cells of close histogenetic origin exhibit a considerably higher phenotypic plasticity within the same developmental germ layer lineage; they can be more rapidly and deeply transdifferentiated into other cell types of the same developmental germ layer lineage without time-consuming and labor-intensive differentiation protocols.

A sufficient body of data pertaining to *in vitro* and *in vivo* transdifferentiation of endodermal cells has been accumulated. Pancreatic ductal cells transplanted into the rat liver differentiate into hepatocytes [84]. Oval cells can also differentiate into endocrine and exocrine pancreatic cells [85]. Islet cells in an *in vitro * culturecan differentiate into hepatocytes if the seeding density increases [87]. Thus, endodermal cells are capable of mutual transdifferentiation and can compensate for the functional insufficiency of another tissue within the endoderm germ layer. However, the problem of shortage of donor material exists both for hepatic and pancreatic cells. For this reason, the search for an optimal source of endodermal cells for substitutive cellular therapy remains rather topical.

Salivary gland cells are one of the potential sources of endodermal cells. The salivary gland is usually formed during the embryonic stage as an ectodermal bud; cells of endodermal origin subsequently migrate into it [88]. Since salivary gland cells are functionally identical to exocrine pancreatic cells, they can be used as a convenient source of endodermal cells for substitutive therapy in patients with hepatic and pancreatic disorders. A sufficiently large body of data pertaining to *in vitro * cultivation of salivary gland cells isolated from humans and animals has been accumulated. The *in vitro * cultured salivary gland cells represent an actively proliferating culture that is capable of undergoing a significant number of passages [89]. Salivary gland cells in humans and animals (mouse, rat, pig) are positive with respect to cytokeratins 18 and 19 and often with respect to α-fetoprotein [90, 91]. Salivary gland cells become capable of synthesizing albumin under certain conditions [92]. However, this source of cellular material remains relatively poorly studied. Thorough elucidation of the mechanisms of hepatocyte differentiation of salivary gland cells and their contribution to the treatment of liver diseases is still to be performed.

**Direct differentiation technique: the use of genetic constructs for somatic cell reprogramming**

The direct technique of cellular differentiation is based on using genetic constructs for the re-programming of various cell types directly into the target cells, bypassing the return to their pluripotent state. One of the major advantages of this approach over using pluripotent ES and iPS cells consists in the absence of risks of teratoma formation. Being a relatively new approach, it requires thorough understanding of the molecular and genetic mechanisms of a certain cellular differentiation and has been recently undergoing active development.

A number of studies have been carried out that demonstrate that direct re-programming of cells of different origins is possible [93]. For instance, functioning β-cells can be obtained from murine exocrine pancreatic cells. The minimum gene set ( *Ngn3,* *Pdx1* and *Mafa* ) required to re-program differentiated cells derived from an adult organism into cells exhibiting the properties of endocrine pancreatic cells has been determined experimentally by the *in vivo* re-expression of key regulatory genes. These cells are identical to endogenous β-cells in terms of their size, shape, and ultrastructure; they express the genes required for β-cell function and can reduce hyperglycemia by actively secreting insulin and facilitating the rearrangement of local blood vessels [94].

As for hepatic cells, there are only very few studies devoted to the obtainment of functionally active hepatocyte-like cells via direct differentiation. This can be mainly attributed to the complexity and multistageness of hepatocyte differentiation, which impedes the search for the key differentiation genes. However, the first success in this area has already been achieved. The lentiviral transfection of 14 genes playing a key role in liver development was used to induce hepatocyte differentiation of fibroblasts obtained from mouse tail-tip [95]. After the analysis of the published data, two gene sets inducing the epithelial phenotype in fibroblasts and expression of hepatocyte markers were selected. The first set consisted of six genes: *Foxa2* , *Foxa3* , *Hnf-1α* , *Hnf-4α* , *Hnf-6* , and *Gata4* ; the second one contained eight genes, including *Foxa1* and *Hlf* [96, 97]. A significant increase in the number of epithelial-like colonies was observed after *Hnf-6* was eliminated from the gene set, whereas elimination of *Hnf-4α* promoted the formation of epithelial-like colonies to an even greater degree. The remaining genes were also divided into two sets: *Gata4* , *Hnf-1α* , *Foxa3* and  *Gata4* , *Hnf-1α* , *Foxa2* ; the former set showed a better result. It is interesting to note that the use of the *Gata4,* *Hnf-1α* and  *Foxa3* gene set provided endogenous Foxa2 and Foxa3 expression, whereas the elimination of any gene from this set blocked hepatocyte re-programming. The induced hepatocyte-like cells were called iHep. These cells were positive with respect to E-cadherin and the tight-junction protein Tjp1. On day 14, 23% of the epithelial-like cells were albumin-positive. iHep were also positive with respect to α-fetoprotein, cytokeratins 18 and 19, Hnf-4α, and cytochromes Р450. No pancreatic differentiation markers were detected; iHep did not exhibit the properties of cell types other than hepatocytes. iHep were also capable of accumulating glycogen and secreting albumin into the medium. An intrasplenic injection of iHep cells to Fah ^-/-^ mice with disturbed tyrosine metabolism, which can survive only if their food contains 2-(2-nitro-4-trifluoromethylbenzene)-1,3-cyclohexandione, resulted in considerable liver re-population (from 5 to 80%). These mice could survive without receiving 2-(2-nitro-4-trifluoromethylbenzene)-1,3-cyclohexandione, whereas an injection of the intact fibroblasts caused death of mice and did not result in liver re-population [95]. All these data attest to the efficiency of the direct differentiation of murine fibroblasts into hepatocyte-like cells via the regulatory factors Gata4, Hnf-1α, and Foxa3. Nevertheless, this approach requires further investigation, since the use of re-programmed fibroblasts is associated with an increased risk of fibrosis formation in the culture. There can be another optimal set of regulatory genes if cells with a minimum tendency to develop fibrosis are used.

Another approach to stimulating liver regeneration is to use genetic vectors carrying the key genes enhancing hepatic cell proliferation ( *Fig. 2* ), reducing apoptosis, or compensating for the gene defects of the liver function [7]. However, this approach requires thorough investigation; including designing optimal and safe vectors for gene transfer, elaborating methods for the delivery of vectors to the liver, etc.

## MOLECULAR AND GENETIC MECHANISMS of hepatocyte differentiation

**Fig. 4:**
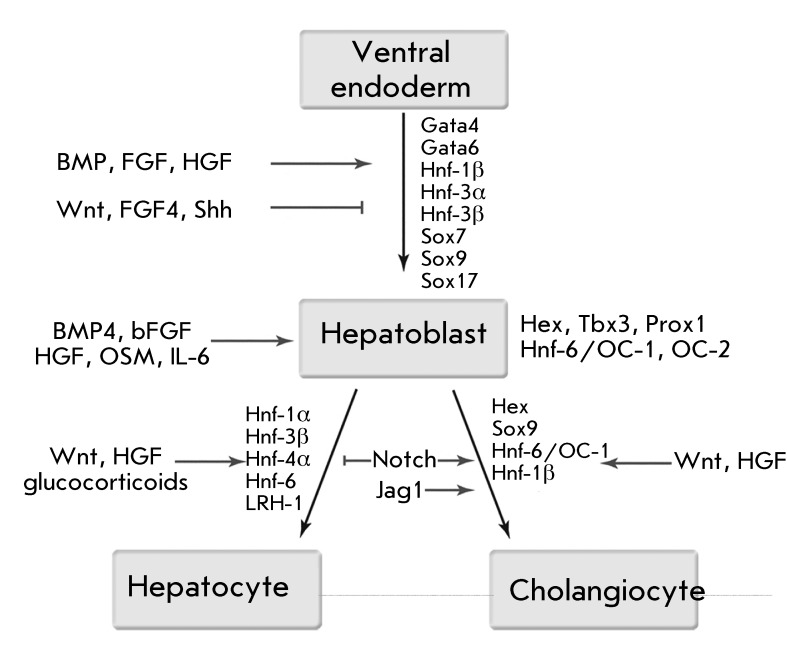
The main stages of liver cell development. Taken and modified from [97, 116].

The definitive endoderm spawns most digestive tract organs, including the liver [53]. Prior to the activation of the organo-specific genes, only several early endoderm markers (including Otx2, Hesx1, Hex, Cdx2) are activated. Mesoendoderm cells in the primitive streak subsequently begin producing a number of factors, such as GSC, Hnf-3β, Cxcr4, Sox17a/b, Brachyury, E-cadherin, VEGER2, VE- cadherin, PDGFRa, Gata4, and Gata6 determining the differentiation of the cells of the definitive endoderm and the mesodermal precursors. The liver emerges from the lateral endoderm of the developing ventral compartment of the fore intestine (approximately at the E8.5 stage of mouse embryo development and week 3 of human pregnancy) [97]. The growth factors secreted by cardiac mesoderm and the mesenchyme of the transverse septum (FGF, BMP) stimulate further differentiation of the underlying endoderm into hepatocyte-like cells. Expression of the *Hnf-3* ( *Foxa* ) genes triggers hepatocyte differentiation in endoderm, which is induced by FGF signals [98]. However, Wnt and FGF4 expression in the mesoderm of the dorsal intestine compartments at this stage inhibits hepatocyte differentiation [99]. Contrariwise, at the late stages (upon formation of hepatocytes and cholangiocytes), Wnt stimulates proliferation and differentiation. HGF, which is required for further growth and proliferation of cells of the liver bud, plays a crucial role for fetal hepatic cells. This type of regulation is performed via the HGF receptor c-Met. HGF impedes hepatoblast commitment into cholangiocytes via blockage of Notch signalling. Endothelial cells have been shown to stimulate liver development (among other factors, due to HGF secretion) [100]. The *Tbx3* gene promotes hepatoblast development via suppression by p19 ^ARF^ [101]. During hepatoblast formation, their shape changes from a cubic to a prolonged one; a pseudo-multilayered epithelium is subsequently formed. This process is regulated by the *Hex * gene. The basement membrane is subsequently destroyed, and the cells proliferate in the surrounding stroma. These and the later morphological changes are regulated by the *Prox1* , *Hnf-6/OC-1* , and *OC-2* genes. Hnf-6 and OC-2 are regulated by E-cadherin, trombospondin-4, and Spp1, which control cell adhesion and migration in a number of tissue types [102]. Notch provides switching of hepatoblast development from the hepatocyte direction towards bile duct formation [103]. Haematopoiesis also plays an important role in the hepatocyte maturation process. After the liver bud begins to protrude from the endodermal canal, haemopoietic cells secreting oncostatin M and IL-6 migrate into it [104]. Oncostatin M stimulates the expression of hepatocyte differentiation markers, induces morphological changes in cells of the liver bud, promotes activation of the synthetic and detoxication properties of the liver, and controls cell adhesion. Glucocorticoids also promote liver maturation and maintain the proliferation and functioning of differentiated hepatocytes. It has been demonstrated that physiological concentrations of dexamethasone (a synthetic glucocorticoid) in fetal liver suppress α-fetoprotein production, initiate albumin synthesis [104], and promote glycogen accumulation [105]. Fig. 4 shows the major stages of development of hepatic cells.

A specific transcription profile that is typical of hepatocytes is maintained by a number of genes, including the *Hnf * family, which encodes hepatocyte nuclear factors. The key genes of this family include *Hnf-1* , a member of the family of POU homeobox genes; *Hnf-3* , a DNA-binding domain; *Hnf-4* , a member of the superfamily of steroid hormones; and *Hnf-6* .

The Hnf-1α and Hnf-1β (or vHnf-1) variants of the Hnf-1 factor interact with homo- or heterodimeric DNA. These proteins have identical DNA-binding domains, but they activate the transcription of different genes. Hnf-1β is expressed in the endoderm of the fore intestine (at stage E5–E6 in mice), whereas Hnf-1α is activated later (at stage E11 in mice), when the parenchyma of the liver is formed. Hnf-1α expression in the fetal liver is lower compared to that of Hnf-1β; however, the Hnf-1α expression level increases after birth. Hnf-1 activates over 1,000 liver-specific genes containing the binding site of this factor in the promoter region; meanwhile, Hnf-1 negatively regulates its own expression. Hnf-4 is a positive regulator of *Hnf-1* , which is capable of activating the expression of this gene; however, the expression of the target genes is independently regulated by these factors [106].

The Hnf-3 subfamily consists of three proteins: Hnf-3α, Hnf-3β , and Hnf-3γ (or Foxa1, Foxa2, and Foxa3, respectively), which bind to monomeric DNA. The members of this subfamily are characterized by strict homology in the area of DNA-binding domains; they can recognize the same nucleotide sequences. Hnf-3α and Hnf-3β regulate gene expression in hepatocytes and in gastric, intestinal, and bronchial epithelium. Hnf-3γ also plays a significant role in gene expression in hepatic, intestinal, and testicular cells. Hnf-3β is formed in the primitive streak on day 7 of mouse embryo development, and Hnf-3α has a similar expression dynamics; however, its concentration is lower. Hnf-3γ expression starts at stage E12 of mouse embryo development.

Hnf-4 consists of three major members (Hnf-4α, Hnf-4β, and Hnf-4γ) and numerous transition variants. Hnf-4 belongs to the superfamily of nuclear steroid hormone receptors; it binds to homodimeric DNA. Hnf-4β has a lower DNA-binding activity and is a weaker transactivator compared to Hnf-4α. Hnf-4α is expressed in the liver, kidney, and pancreas. Hnf-4β is also expressed in these organs, as well as in the stomach, intestine, lungs, ovaries, and testicles, whereas Hnf-4γ is expressed in the kidney, pancreas, testicles, but not in the liver. Hnf-4 is the key regulator of tissue-specific gene expression in visceral endoderm, which is required for normal expression of the secreted factors, such as α-fetoprotein, apolipoproteins, the retinol-binding protein, etc. Some researchers believe that Hnf-4α plays a key role by triggering a reaction cascade and maintaining hepatocyte-specific transcription. Hnf-4α binds to approximately 12% of the genes expressed in hepatocytes, whereas the other transcription factors can bind to no more than 2.5% of the promoter regions [106]. Being one of the earliest endodermal markers, Hnf-4α emerges in mouse embryos on day 5 of development. Prior to stage E9, Hnf-4α expression is confined to the extra-embryonic visceral endoderm; then it is formed in the liver and intestine. In an adult organism, Hnf-4α is expressed in the liver, kidney, intestine, and pancreas.

Hnf-6 belongs to the family of Onecut transcription factors (also known as OC-1). Hnf-6 binds to the CREB-binding protein (CBP) and is expressed in the liver, pancreas, and the nervous system. Hnf-6 is detected on day 6 of fetal development. Between days 12.5 and 15 it disappears from the mouse embryonic liver to emerge there again after day 15. In an adult organism, Hnf-6 is expressed in the liver, pancreas, encephalon, and testicles. It is an interesting fact that Hnf-3β and Hnf-6 potentially regulate the expression of the same genes; they are crucial to hepatocyte functioning. Hnf-6 recognizes the –138 to –126 region of the *Hnf-3β * promoter; it is required to activate this promoter. Meanwhile, Hnf-3β is capable of binding to the Hnf-6 promoter and represses it [107]. Hnf-6 promotes hepatoblast differentiation into biliary duct cells, whereas Hnf-3β plays a key role in hepatocyte differentiation and functioning.

**Fig. 5:**
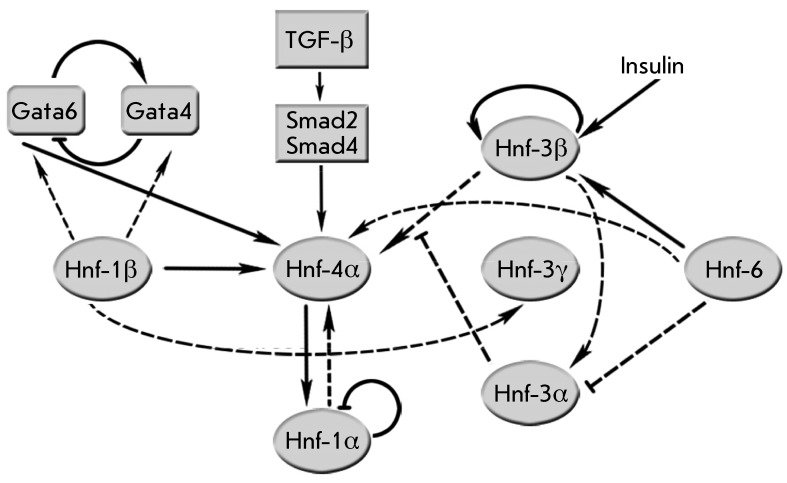
Transcriptional hierarchy of hepatocyte nuclear factors. The interconnections possessing the most universal character are denoted by continuous lines; the dotted lines show the regulation revealed at certain stages of development. Taken from [108].

Expression of nuclear hepatocyte factors and other markers of visceral endoderm during the fetal development is induced by Gata6. TGF-β family growth factors are also capable of inducing the expression of these markers. The hypothetical regulatory pathways ensuring the maintenance of the pattern of liver-specific gene expression are shown in Fig. 5 [108].

## PROBLEMS AND PROSPECTS

One of the major problems emerging upon transplantation of donor hepatocytes is poor engraftment and elimination by the immune system within several months after the transplantation. The extracellular matrix has been shown to play an important role in the survival and engraftment of transplanted cells. Either collagen or fibronectin was intraportally injected into rat liver prior to the transplantation of donor cells in a study investigating the effect of the extracellular matrix on the engraftment of donor hepatocytes. Four days following the transplantation, the percentage of hepatocytes that survived in the liver had increased more than tenfold when either collagen or fibronectin was preliminarily introduced [109]. Simultaneous introduction of hepatocytes and transformation growth factors (TGF-α) can also increase the number of survived cells [110]. Cell survival is known to depend on the presence of a corresponding “cellular niche.” Temporary blockage of the portal vein inducing ischemia and the partial death of hepatic cells was used in experiments on macaque monkeys. Upon subsequent transplantation of donor hepatocytes, their percentage in the recipient’s liver was about 10% of its weight [111]. However, it remains disputable whether it is reasonable to use this approach in clinical practice.

In order to increase the lifespan of transplanted hepatocytes in a patient’s organism, ABO and HLA compatible donors are selected. Immunosuppression is now frequently achieved using anti-IL-6 monoclonal antibodies in combination with small doses of the drugs tacrolimus and sirolimus [112]. Natural killer (NK) cells play an important role in the elimination of introduced hepatocytes. It has been shown that blockage of NK cells in the liver by specific or local immunosuppression enhances the survival rate and proliferation of the transplanted hepatocytes [113]. Moreover, the use of hepatic stem cells can have a longer-term effect due to the fact that frequently stem cells are not eliminated by the recipient’s immune system and can maintain their population for a long time, giving rise to a population of differentiated cells.

Improving the quality of the isolated hepatocytes is another topical problem. The use of a Celsior ^® ^ solution to store and transport liver bioptates prevents hepatocyte degradation and death [22]. Perfusion of the donor tissue with N-acetylcysteine has also made it possible to improve the quality of the isolated cells [114].

Biological safety and efficacy remain the key issues in modern cellular therapy. A reliable protocol for the elimination of undifferentiated cells from the transplant is required when pluripotent (ES and iPS) cells are used, since pluripotent ES and iPS cells are capable of teratoma formation. The assessment of the risk of fibrosis formation when certain cell types are used remains problematic, as well. The percentage of donor MSC and their degree of hepatocyte differentiation have been determined in a study of the ability of bone marrow MSC to restore hepatic cells upon acute and chronic lesions. The contribution of the donor cells four weeks following the transplantation turned out to be low (about 0.08% and 3–4% of the total number of hepatic cells upon acute and chronic lesion, respectively); only 5–10% of them had a hepatocyte phenotype. A significant percentage of donor cells (about 35%) had a myofibroblast phenotype; most of the cells resided within septal fibrosis areas [78]. It becomes obvious that a quantitative assessment of the efficiency of hepatocyte differentiation is required prior to using certain cell types, as well as elucidating whether there is a risk in fibrosis formation by these cells. One of the key tasks of cellular biology is to search for an available source of cells with a low pro-fibrogenic potential and high hepatocyte differentiation ability. Moreover, there should be an opportunity to use these cells for both allogenic and autologous transplantation.

Direct cell differentiation seems to have a high potential; however, one needs a thorough understanding of the molecular mechanisms of the processes occurring upon hepatocyte development and differentiation to elaborate standard protocols. One of the key tasks in this field is to determine the key differentiation genes that would be optimal for the transdifferentiation of cells of various histogenetic origins.

## CONCLUSIONS

A number of fundamentally different approaches to the therapy of liver disorders are currently being developed. Various cell types are being tested *in vitro* and *in vivo* , and the optimal differentiation procedures are being selected. Despite some encouraging results obtained on laboratory animals, a sufficiently safe and efficient method is still to be found. A shortage of donor liver and donor hepatocytes stimulates the search for alternative sources of cellular material; however, no cells that could be able to perform hepatocyte functions to an adequate degree have been obtained thus far. A search for the optimal cell type and development of differentiation procedures that would satisfy the biological safety and functional efficiency criteria is needed. 

## Abbreviations

ES cells: embryonic stem cells
iPS cells: induced pluripotent stem cells
HSC: haematopoietic stem cells, MSC – mesenchymal stem cells
SP cells: side population cells
